# Relationship between Antibiotic Resistance, Biofilm Formation, and Biofilm-Specific Resistance in *Acinetobacter baumannii*

**DOI:** 10.3389/fmicb.2016.00483

**Published:** 2016-04-12

**Authors:** Lihua Qi, Hao Li, Chuanfu Zhang, Beibei Liang, Jie Li, Ligui Wang, Xinying Du, Xuelin Liu, Shaofu Qiu, Hongbin Song

**Affiliations:** Department of Infectious Disease Control, Institute of Disease Control and Prevention, Academy of Military Medical SciencesBeijing, China

**Keywords:** *Acinetobacter baumannii*, biofilm, antibiotic resistance, biofilm-specific resistance, pulsed-field gel electrophoresis

## Abstract

In this study, we aimed to examine the relationships between antibiotic resistance, biofilm formation, and biofilm-specific resistance in clinical isolates of *Acinetobacter baumannii*. The tested 272 isolates were collected from several hospitals in China during 2010–2013. Biofilm-forming capacities were evaluated using the crystal violet staining method. Antibiotic resistance/susceptibility profiles to 21 antibiotics were assessed using VITEK 2 system, broth microdilution method or the Kirby-Bauer disc diffusion method. The minimum inhibitory concentration (MIC) and minimum biofilm eradication concentration (MBEC) to cefotaxime, imipenem, and ciprofloxacin were evaluated using micro dilution assays. Genetic relatedness of the isolates was also analyzed by pulsed-field gel electrophoresis (PFGE) and plasmid profile. Among all the 272 isolates, 31 were multidrug-resistant (MDR), and 166 were extensively drug-resistant (XDR). PFGE typing revealed 167 pattern types and 103 clusters with a similarity of 80%. MDR and XDR isolates built up the main prevalent genotypes. Most of the non-MDR isolates were distributed in a scattered pattern. Additionally, 249 isolates exhibited biofilm formation, among which 63 were stronger biofilm formers than type strain ATCC19606. Population that exhibited more robust biofilm formation likely contained larger proportion of non-MDR isolates. Isolates with higher level of resistance tended to form weaker biofilms. The MBECs for cefotaxime, imipenem, and ciprofloxacin showed a positive correlation with corresponding MICs, while the enhancement in resistance occurred independent of the quantity of biofilm biomass produced. Results from this study imply that biofilm acts as a mechanism for bacteria to get a better survival, especially in isolates with resistance level not high enough. Moreover, even though biofilms formed by isolates with high level of resistance are always weak, they could still provide similar level of protection for the isolates. Further explorations genetically would improve our understanding of these processes and provide novel insights in the therapeutics and prevention against *A. baumannii* biofilm-related infections.

## Introduction

*Acinetobacter baumannii* is an important nosocomial pathogen that is responsible for a wide range of human infections, resulting in high levels of morbidity and mortality (Bergogne-Berezin and Towner, 1996). Due to the high prevalence of multidrug-resistant (MDR) and extensively drug-resistant (XDR) isolates, *A. baumannii* has been identified among the top seven pathogens threatening our healthcare-delivery system and as a prime example of unmet medical need (Talbot et al., 2006). Antibiotic resistance is primarily the consequence of genetic transfer of resistance genes via plasmids, and the mutation of target genes (Andersson and Hughes, 2010). Given the tremendous capacity for acquiring antibiotic resistance determinants, *A. baumannii* may be leaving us few effective therapeutic options (Perez et al., 2008). Moreover, this is not the only reason antibiotics fail, and for many occasions, it may not even be the main reason (Levin and Rozen, 2006). In fact, biofilm formation is another effective way for bacteria to survive in the presence of antibiotics (Hall-Stoodley et al., 2004), especially for *A. baumannii* which is one of the most common bacterial causes of biofilm-related contamination of medical devices (Singhai et al., 2012).

Biofilms are assemblages of microorganisms, encased in a matrix, that function as a cooperative consortium. Biofilm mode of life is a feature common to most microorganisms in natural and medical systems which constitutes a protected mode of growth that allows survival in hostile environments (McDougald et al., 2012). Biofilm-specific resistance has been reported to be significantly higher than antibiotic resistance of planktonic bacteria (Hoyle and Costerton, 1991). Therefore, biofilm-related infections are more difficult to clear and more prone to relapse (Cerqueira and Peleg, 2011). The connection between biofilm and antibiotic resistance is of considerable interest to biomedical researchers. Notably, several studies have demonstrated that low doses of certain antibiotics can induce biofilm formation (Hoffman et al., 2005; Kaplan, 2011), indicating that biofilm regulation may be involved in the global response to external stresses, including antibiotics (Kaplan, 2011). However, it is currently unclear whether there is a quantitative correlation between biofilm formation and antibiotic resistance. Over the past two decades, multiple studies have yielded conflicting results. For example, while Abidi et al. studied 22 *Pseudomonas aeruginosa* isolates and concluded that biofilm production was significantly higher in MDR isolates (Abidi et al., 2013), Atashili et al. failed to find a significant difference in biofilm formation among MDR and non-MDR *Staphylococcus aureus* (Atashili et al., 2014). For *A. baumannii*, while Gurung et al. studied 60 isolates and found a positive relationship between biofilm formation and antibiotic resistance (Gurung et al., 2013), Perez studied 116 isolates and detected an inverse one between meropenem resistance and biofilm production (Perez, 2015). Additionally, not enough reports have analyzed the quantitative correlation between biofilm-specific resistance and antibiotic resistance/biofilm forming capacity, and the enhancement in resistance after biofilm formation has not been quantified either.

In this study, we examined antibiotic resistance, biofilm formation, and biofilm-specific resistance in 272 clinical *A. baumannii* isolated from patients in China. Our results not only highlight the elegant balance between antibiotic resistance and biofilm formation developed by this organism to enhance its viability, but also provide information about biofilm-specific resistance that is expected to help understanding the role of biofilm in resistance and contribute to resolving the problem in treatment of biofilm-related infection.

## Materials and methods

### Bacterial strains and growth conditions

A total of 272 strains were collected from several general hospitals in China during 2010–2013. The collection and use of clinical isolated strains was approved by Institutional review board (IRB) of Academy of Military Medical Sciences, China. All strains were capable of growth at 44°C, and were identified as *A. baumannii* using API 20 NE tests (bio-Mérieux, Marcy l'Etoile, France). The type strain of *A. baumannii* (ATCC 19606) was used for comparison in biofilm assay (Lee et al., 2008). Strains were stored as glycerol stocks at −80°C, and were cultivated in nutrient agar at 37°C for 18–20 h without shaking for further analysis.

### Pulsed-field gel electrophoresis (PFGE) and plasmid profile

The genetic diversity and relatedness of the isolates was analyzed by PFGE as previously described (Seifert et al., 2005) and plasmid profile. Briefly, pure bacterial cultures were embedded in agarose plugs and incubated in lysis buffer followed by digestion with 20 mg/mL proteinase K. The plugs were thoroughly washed and then digested for 3 h with *Apa I* restriction endonuclease (TaKaRa, Dalian, Beijing, China). DNA separation was performed in 0.5 × TBE buffer in a pulsed-field electrophoresis system (Chef Mapper; Bio-Rad Laboratories, Hercules, CA, USA) with the following conditions: temperature 14°C; voltage 6 V/cm; switch angle 120°; switch ramp 5–20 s for 19 h. The size standard strain *Salmonella enterica* serotype Braenderup H9812 was digested with *Xba*I. The interpretation of the PFGE banding patterns was performed with BioNumerics software version 6.0 (Applied Maths, Sint-Martens-Latem, Belgium). A tree indicating relative genetic similarity was constructed based on the unweighted pair group method of averages and a position tolerance of 1.5%. Band patterns indistinguishable from each other were defined as one PFGE pattern type and clusters were defined as isolates with band patterns of 80% similarity or above.

For plasmid profile, the plugs were performed with restriction endonuclease digestion S1 for 10min at 37°C, and pulse time ramped from 5 to 20 s at 6.0 V/cm for 12 h.

### Antimicrobial susceptibility testing

Antibiotic resistance to 21 drugs covering all the nine antimicrobial categories including aminoglycosides, carbapenems, fluoroquinolones, penicillins + β-lactamase inhibitors, folate pathway inhibitors, tetracyclines, penicillins, cephems, and lipopeptides was assessed for each isolate. These nine antimicrobial categories were epidemiologically significant ones constructed for *Acinetobacter* spp. to determine MDR/XDR in this species (Magiorakos et al., 2012).

Minimum inhibitory concentrations (MICs) to 14 drugs including gentamicin, tobramycin, amikacin, imipenem, meropenem, ciprofloxacin, levofloxacin, ampicillin/sulbactam, piperacillin/tazobactam, ceftazidime, ceftriaxone, cefepime, trimethoprim/sulfamethoxazole, and piperacillin were assessed on VITEK 2 system (bioM'erieux, Marcy l'Etoile, France) using software version 5.04 and AST-GN09 test card, according to the manufacturer's instructions. Interpretive breakpoints for susceptible, intermediate and resistant were consistent with Clinical and Laboratory Standards Institute guidelines (CLSI, 2015). MICs to three drugs including polymyxin B, doxycycline and minocycline were assessed using broth microdilution method and resistance to four drugs including cefotaxime, tetracycline, mezlocillin, and ticarcillin/clavulanic acid were assessed using the standard disc diffusion method (Oxoid, Hampshire, UK). These results were interpreted according to CLSI (2015). Strains non-susceptible to at least one agent in three or more antimicrobial categories were defined as MDR, while those non-susceptible to at least one agent in all but two or fewer antimicrobial categories were considered as XDR (Magiorakos et al., 2012). In contrast, those resistant to 0–2 antimicrobial categories were described separately and referred to as non-MDR for the correlation analyses between antibiotic resistance and biofilm formation.

### Biofilm formation assays

The biofilm formation capacity of each strain was estimated using the crystal violet staining method described previously (O'Toole, 2011), with minor adjustment. Briefly, strains were cultured in nutrient agar for 18–20 h and adjusted to 0.5 McFarland units (~1.5 × 10^8^ CFU/mL) with 0.85% NaCl medium. A 10-μL aliquot of each suspension was then diluted 1:20 in 190 μL of fresh Luria-Bertani (LB) medium in 96-well polyvinyl chloride microtiter plates. After incubation at 37°C for 24 h, the plates were washed three times with 0.85% NaCl medium, and each well was stained with 200 μL of 0.1% crystal violet (CV) for 20 min at ambient temperature. The plates were again washed three times to remove excess stain, and the remaining CV was solubilized by incubating with 200 μL of 95% ethanol for 20 min. The optical density at 550 nm (OD_550_) of each well was then measured (multi scan MS352, Thermo Labsystems), to obtain the biofilm formation capacity of the isolate. ATCC 19606 was used as a reference strain, while un-inoculated LB medium was used as a negative control. All experiments were carried out in triplicate.

Since there is no universally recognized reference value used for evaluating biofilm formation capacity (Mendoza-Olazarán et al., 2014), in this study, strains with OD_550_ values greater than that of the negative control were considered positive for biofilm formation. Specifically, those with OD_550_ values greater than that of the negative control, but less than that of the reference strain (ATCC 19606) were characterized as weak biofilm formers, while those with OD_550_ values greater than that of ATCC19606 were considered strong biofilm formers.

### Growth rate analysis

The growth of 12 strong and 12 weak biofilm formers were measured (Hung et al., 2012). Strains were cultured in nutrient agar for 18–20 h and adjusted to 0.5 McFarland units with 0.85% NaCl medium, and diluted 1: 20 in LB medium. Growth curves were performed in triplicate, incubating for 24 h at 37°C with shaking at 200 rpm. Bacterial growth was monitored by measuring the OD_600_ values of the culture.

### Biofilm susceptibility test

The minimum biofilm eradication concentrations (MBECs) of cefotaxime, imipenem and ciprofloxacin for 31 isolates were assessed using a microdilution assay adapted from that described by Ceri et al. (2001). The 31 strains were selected using a systematic sampling method according to their biofilm formation capacities. The test strains were cultivated in 96-well plates for 24 h at 37°C, as described above, to allow for biofilm formation. Biofilms were then treated with 256–524,288 μg/mL cefotaxime, 2–4096 μg/mL imipenem, and 4–8192 μg/mL ciprofloxacin respectively for 24 h at 37°C, rinsed, and incubated with fresh LB medium for 24 h at 37°C to allow for recovery. The minimum antibiotic concentration at which no viable cell counts were recovered from the biofilm material (OD_600_ < 0.1) was considered the MBEC. All assays were repeated in triplicate.

### Statistical analyses

OD values were expressed either as means ± standard deviations (SDs) or as median values (interquartile range, IQR) based on the distribution and the homogeneity of variance. Spearman's rank correlation tests was used for intergroup comparisons, specifically, comparison of biofilm formation among isolates susceptible, resistant to one, two antimicrobial categories, MDR and XDR, among isolates susceptible, intermediate and resistant to each antibiotic, comparison of antibiotic resistance/biofilm formation among isolates with different plasmid profiles, as well as correlations between MICs and MBECs, MICs and biofilm formation, and enhancement in resistance after biofilm formation and the level of biomass produced. Wilcoxon rank sum test was used for comparison of biofilm formation between isolates susceptible/non-susceptible to each antimicrobial category. Data analyses were performed using SPSS for Windows version 19.0 (SPSS Statistics, Inc., Chicago, IL, USA). *P* < 0.05 was considered statistically significant for all tests.

## Results

### Antimicrobial susceptibility testing

**Figure 1:**
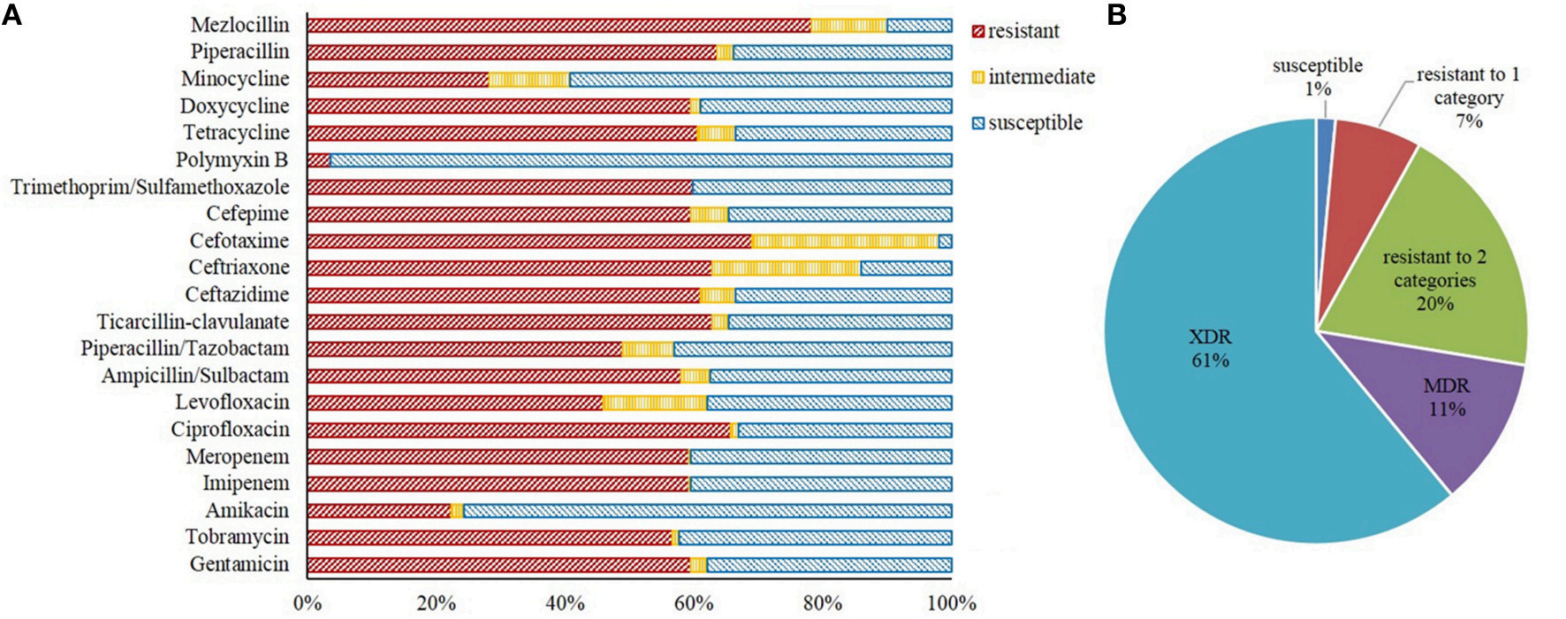
**Antibiotic resistance phenotypes of *A. baumannii* isolates examined in this study. (A)** The resistance rate of all strains to 16 of the 21 antibiotics were above 50%. **(B)** Approximately 72.4% of the isolates exhibited multidrug or extensively drug resistance.

Resistance to mezlocillin was the most common (213, 78.3%), followed by cefotaxime (188, 69.1%), ciprofloxacin (179, 65.8%), piperacillin (173, 63.6%), ticarcillin/clavulanic acid (171, 62.9%), ceftriaxone (171, 62.9%), ceftazidime (166, 61.0%), tetracycline (165, 60.7%), trimethoprim/sulfamethoxazole (163, 59.9%), gentamicin (162, 59.6%), cefepime (162, 59.6%), doxycycline (162, 59.6%), imipenem (161, 59.2%), meropenem (161, 59.2%), ampicillin/sulbactam (158, 58.1%), tobramycin (154, 56.6%), piperacillin/tazobactam (133, 48.9%), levofloxacin (125, 46.0%), minocycline (77, 28.3%), amikacin (61, 22.4%), and polymyxin B (ten, 3.7%). Approximately 60% of the strains were resistant to at least one of the carbapenems antibiotics tested, which included imipenem and meropenem here (Figure 1A).

No isolate was resistant to all of the 21 antibiotics. Of the 272 *A. baumannii* isolates tested, 268 were resistant to at least one antibiotic. Specifically, 18 and 53 isolates were resistant to only one or two of the 21 antibiotics tested, respectively, 31 were classified as MDR, and 166 were classified as XDR. As such, ~72.4% of the 272 isolates were either MDR or XDR (Figure 1B).

### Biofilm formation and growth rate analysis

**Figure 2:**
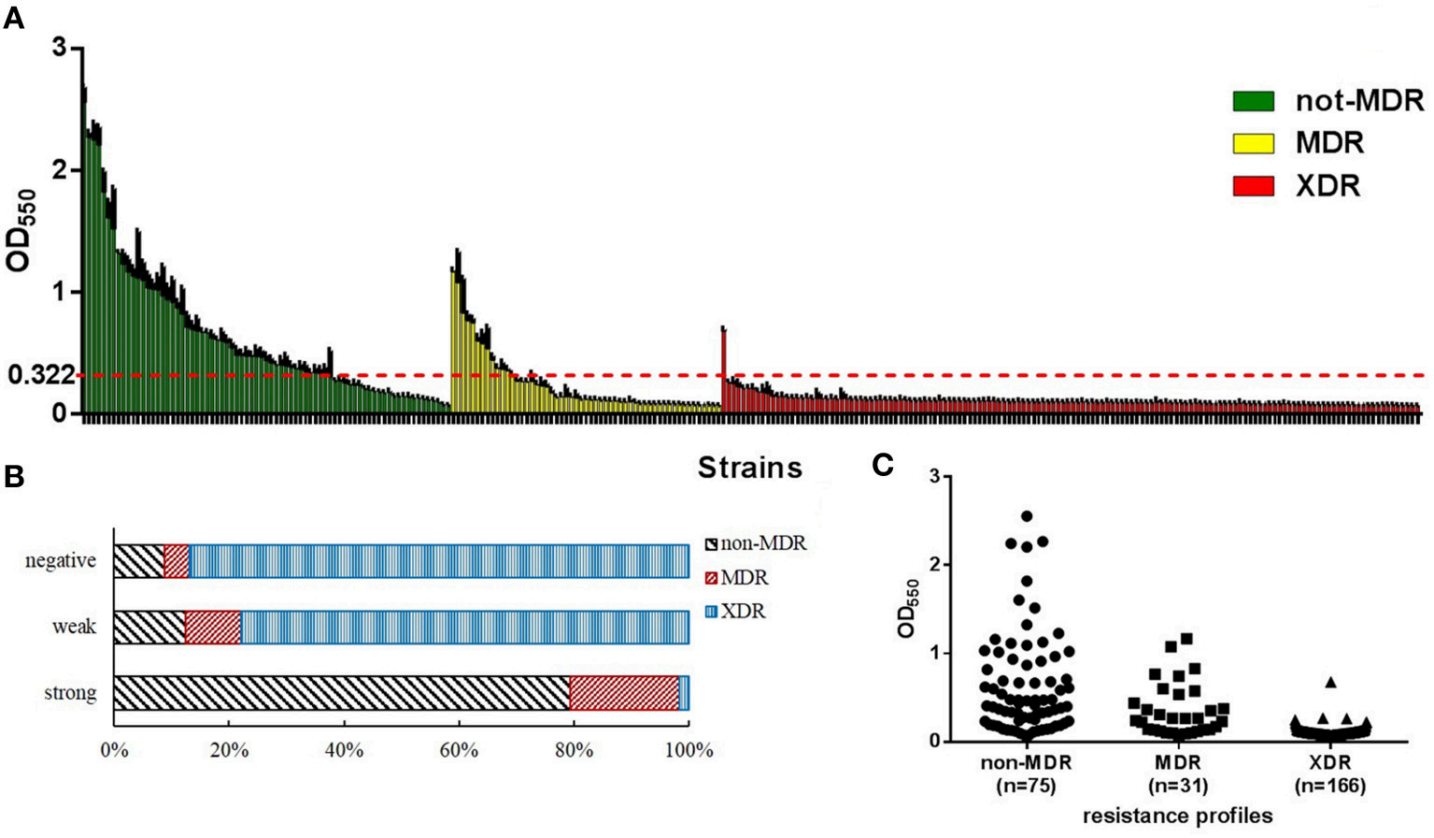
**Biofilm formation of *A. baumannii* isolates examined in this study**. OD_550_, optical density at 550 nm, represents biofilm forming capacity. **(A)** The level of biofilm formation was assessed for each isolate. Strains were divided into three groups according to their antibiotic resistance phenotypes: non-multidrug-resistant (non-MDR), multidrug-resistant (MDR), and extensively drug-resistant (XDR). The red line shows the optical density at 550 nm (OD_550_) of the biofilm biomass produced by the reference strain (ATCC 19606). Larger proportion of non-MDR isolates tended to form stronger biofilms (higher OD_550_ values). **(B)** Distribution of resistance phenotypes among different biofilm production capacities displayed as a percentage stacked bar graph. Population that exhibited more robust biofilm formation likely contained larger proportion of non-MDR isolates. **(C)** Distribution of biofilm formation of isolates with different resistance phenotypes. Isolates with higher level of resistance tended to form weaker biofilms.

The biofilm-forming capacity of each isolate is summarized in Figure 2A. The OD_550_ values for the reference strain (ATCC19606) and negative control were 0.322 ± 0.048 and 0.080 ± 0.001, respectively. The OD_550_ values for the clinical isolates ranged from 0.078 ± 0.003 to 2.556 ± 0.137. The resulting distribution was positively skewed, and the median value (IQR) was 0.118 (0.095, 0.270). In total, 249 (91%) isolates were positive for biofilm formation, and 63 (23%) isolates exhibited more robust biofilm formation than ATCC 19606. No significant difference in growth rate of the strong and weak biofilm formers was observed (Figure S3) indicating that the difference in biofilm formation was not due to the growth rate.

### Genotyping profiles

PFGE typing revealed that apart from two isolates which were not typeable, there were 167 pattern types among the remaining 270 isolates. With a similarity of 80%, all isolates formed a total of 103 clusters (Figure S1). One-hundred-and-fifty of the 197 MDR/XDR strains were classified into the only eight clusters containing more than five isolates: cluster4 (*n* = 16), cluster6 (*n* = 27), cluster7 (*n* = 20), cluster9 (*n* = 48, including two non-MDR isolates) cluster10 (*n* = 17), cluster11 (*n* = 13), cluster22 (*n* = 6), and cluster34 (*n* = 6, including one non-MDR isolate). The remaining 23% of the MDR/XDR and 97% of the non-MDR isolates were distributed in a scattered pattern. Such results indicated a high genetic diversity among the *A. baumannii* strains in China, meanwhile MDR and XDR isolates built up the main prevalent genotypes.

Plasmids were detected in 255 (93.8%) isolates, among which 152 isolates harbored one plasmid, 58 isolates harbored two, 36 isolates harbored three, four isolates harbored four, one isolate harbored five, one isolate harbored six, and three isolates harbored seven. The plasmids ranged from 5.28 to 207.6 kbp in size. Resistant isolates were more likely harboring more plasmids (*P* < 0.05). But no significant correlation were found between biofilm forming capacity and plasmid harbored (*P* > 0.05). Strains with the same plasmid profile could have obviously different resistance phenotypes and biofilm forming capacities, and strains with different plasmid profiles may have the same resistance phenotype and biofilm forming capacity (Figure S2).

### Antibiotic-susceptible isolates tended to form stronger biofilms than resistant strains

To explore if there's any correlation between biofilm formation and antibiotic resistance, we first analyzed the composition of the biofilm formation groups with respect to resistance phenotypes. Among the 63 strong biofilm-formers, 79.4% were non-MDR isolates and, 20.6% were MDR/XDR ones. The 186 weak biofilm-formers consisted of 12.4% non-MDR and 87.6% MDR/XDR isolates. The 23 strains that were negative for biofilm formation consisted of 8.7% non-MDR and 91.3% MDR/XDR isolates (Figure 2B). These constituent ratios revealed that the population that exhibited more robust biofilm formation likely contained larger proportion of non-MDR isolates The Spearman's correlation coefficient (*r*_s_) for this comparison was −0.601 (*P* < 0.001).

Next we found that non-MDR *A. baumannii* isolates tended to form stronger biofilms than MDR and XDR strains; this conclusion was also confirmed by statistical analyses (*r*_s_ = −0.720, *P* < 0.001; Table 1, Figure 2C), indicating a negative correlation between biofilm formation capacity and antibiotic resistance phenotypes. Non-MDR isolates were of greater possibility to be strong biofilm producers than MDR or XDR ones. Considered that a majority of the MDR/XDR strains belonged to eight main PFGE clusters and they were all weak or negative in biofilm formation, we analyzed the dataset excluding the isolates in these clusters in case they had an impact on the result. But we found the correlation between biofilm formation and resistance phenotypes still exist (*r*_s_ = −0.468, *P* < 0.001).

**Table 1:** **Biofilm forming capacities of *A. baumannii* with different antibiotic resistance phenotypes**.

**Resistance phenotype**	**N**	OD_550_^*^	***r*_*s*_**	***P*-value**
Non-MDR	75	0.439 (0.234, 0.890)	−0.720	<0.001
MDR^**^	31	0.266 (0.139, 0.488)		
XDR^**^	166	0.100 (0.087, 0.117)		

* OD_550_, optical density at 550 nm; data shown in median (interquartile range, IQR);

** MDR, multidrug-resistant; XDR, extensively drug-resistant.

**Figure 3:**
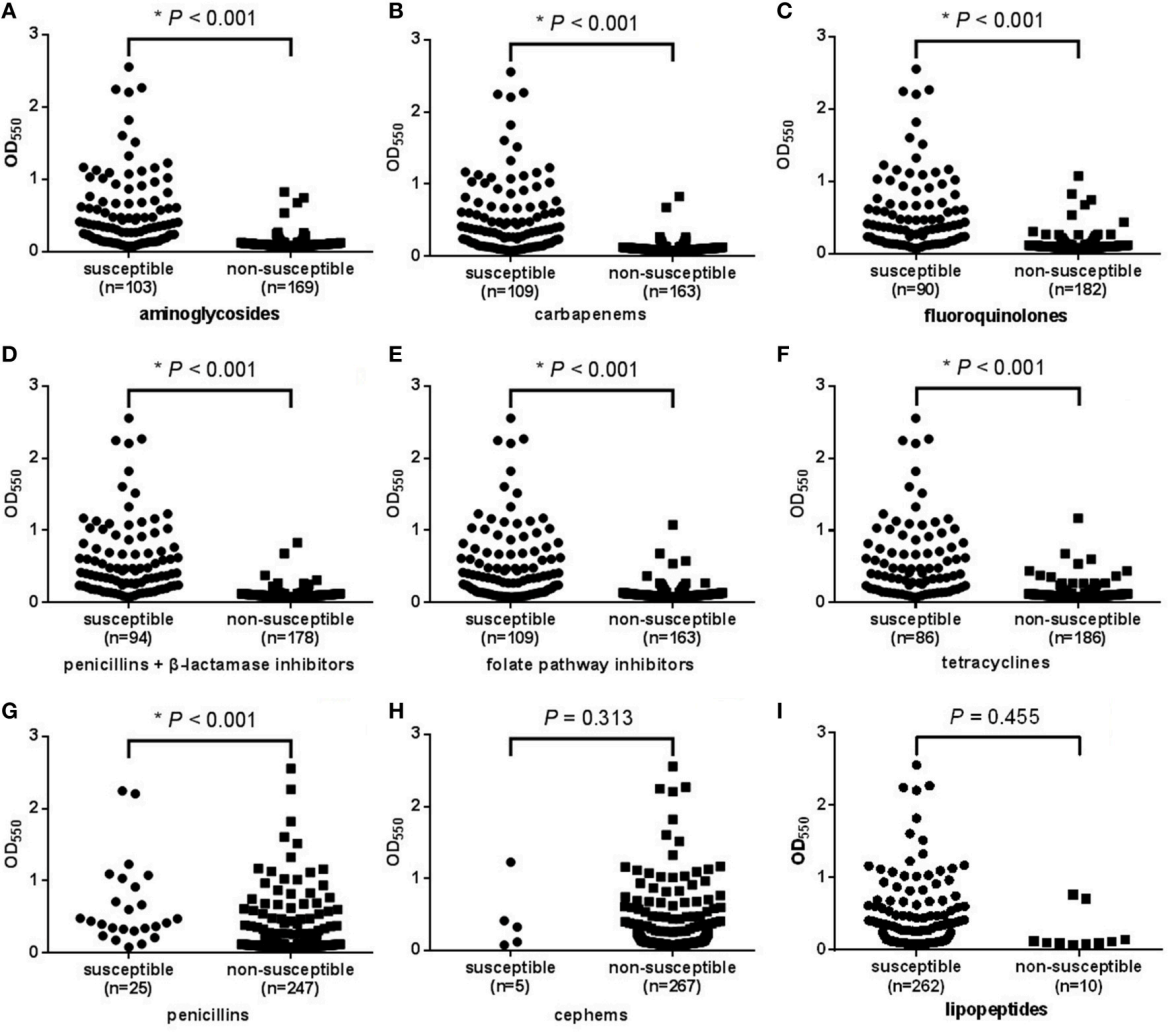
**Relationship between biofilm formation and the resistance of *A. baumannii* isolates to each of the nine antimicrobial categories**. OD_550_, optical density at 550 nm, represents biofilm forming capacity. **(A–G)**: For aminoglycosides, carbapenems, fluoroquinolones, penicillins + β-lactamase inhibitors, folate pathway inhibitors, tetracyclines, and penicillins, susceptible isolates tended to form stronger biofilms (higher OD_550_ values) than non-susceptible ones. **(H,I)**: For cephems and lipopeptides, no significant difference in biofilm formation among susceptible and non-susceptible isolates was observed.

Finally, to determine whether biofilm formation is correlated with resistance to any particular antibiotic(s), we compared the biofilm forming capacities among strains with different resistance profiles to each of the 21 antibiotics. The results revealed that apart from polymyxin B, for each antibiotic, susceptible isolates could form stronger biofilms than intermediate and resistant ones, meaning a negative correlation between biofilm quantity and resistance profile to each of the 20 antibiotics (*r*_s_ = 0.284–0.730, *P* < 0.001; Table 2). For polymyxin B, no significant correlation was observed (*r*_s_ = 0.046, *P* = 0.455; Table 2). The correlation between biofilm and resistance to the nine antimicrobial categories was analyzed as well. For seven of the categories including aminoglycosides, carbapenems, fluoroquinolones, penicillins + β-lactamase inhibitors, folate pathway inhibitors, tetracyclines, and penicillins, susceptible isolates could form stronger biofilms than non-susceptible ones (*P* < 0.001; Figures 3A–G). While for cephems and lipopeptides, no significant difference in biofilm formation between susceptible and non-susceptible isolates was observed (Figures 3H,I). We supposed this was because of the huge difference in sample size. For cephems, 267 isolates were non-susceptible and only five were susceptible. For lipopeptides, which also means polymyxin B mentioned above, 262 isolates were susceptible and only 10 were non-susceptible.

**Table 2:** **Correlation between the level of biofilm formation and resistance to 21 antibiotics in *A. baumannii* clinical isolates**.

**Antimicrobial category**	**Antimicrobial agent**	**OD**_550_^*^			***r*_*s*_**	***P*-value**
		S^**^	I^**^	R^**^		
Aminoglycosides	Gentamicin	0.366 (0.177, 0.687)	0.090 (0.084, 0.109)	0.100 (0.089, 0.119)	−0.647	<0.001
	Tobramycin	0.329 (0.138, 0.643)	0.745 (0.416, 0.788)	0.100 (0.089, 0.118)	−0.603	<0.001
	Amikacin	0.129 (0.097, 0.392)	0.105 (0.088, 0.109)	0.105 (0.087, 0.122)	−0.284	<0.001
Carbapenems	Imipenem	0.360 (0.156, 0.679)	0.117 (0.117, 0.117)	0.100 (0.088, 0.117)	−0.674	<0.001
	Meropenem	0.360 (0.156, 0.679)	0.125 (0.125, 0.125)	0.100 (0.088, 0.117)	−0.674	<0.001
Fluoroquinolones	Ciprofloxacin	0.398 (0.200, 0.751)	0.440 (0.374, 0.758)	0.100 (0.088, 0.120)	−0.690	<0.001
	Levofloxacin	0.372 (0.187, 0.700)	0.108 (0.088, 0.124)	0.099 (0.088, 0.113)	−0.662	<0.001
Penicillins + β-lactamase inhibitors	Ampicillin/sulbactam	0.380 (0.200, 0.704)	0.112 (0.097, 0.132)	0.100 (0.088, 0.117)	−0.709	<0.001
	Piperacillin/tazobactam	0.337 (0.139, 0.666)	0.110 (0.094, 0.120)	0.103 (0.087, 0.119)	−0.573	<0.001
	Ticarcillin/clavulanic acid	0.412 (0.233, 0.761)	0.102 (0.097, 0.115)	0.100 (0.088, 0.119)	−0.705	<0.001
Cephems	Ceftazidime	0.400 (0.204, 0.737)	0.231 (0.132, 0.422)	0.100 (0.087, 0.116)	−0.730	<0.001
	Ceftriaxone	0.359 (0.187, 0.652)	0.400 (0.234, 0.846)	0.100 (0.088, 0.117)	−0.704	<0.001
	Cefotaxime	0.329 (0.121, 0.417)	0.400 (0.218, 0.824)	0.103 (0.089, 0.124)	−0.625	<0.001
	Cefepime	0.404 (0.231, 0.761)	0.094 (0.085, 0.118)	0.101 (0.088, 0.119)	−0.673	<0.001
Folate pathway inhibitors	Trimethoprim/sulfamethoxazole	0.342 (0.139, 0.682)	−	0.103 (0.090, 0.121)	−0.556	<0.001
Lipopeptides	Polymyxin B	0.118 (0.095, 0.269)	−	0.105 (0.092, 0.138)	−0.046	0.455
Tetracyclines	Tetracycline	0.395 (0.177, 0.793)	0.095 (0.085, 0.112)	0.137 (0.089, 0.124)	−0.574	<0.001
	Doxycycline	0.378 (0.177, 0.704)	0.188 (0.130, 0.275)	0.100 (0.087, 0.118)	−0.688	<0.001
	Minocycline	0.205 (0.010, 0.478)	0.099 (0.085, 0.116)	0.105 (0.089, 0.121)	−0.424	<0.001
Penicillins	Piperacillin	0.389 (0.208, 0.696)	0.535 (0.288, 0.840)	0.100 (0.088, 0.118)	−0.724	<0.001
	Mezlocillin	0.474 (0.336, 0.940)	0.407 (0.172, 0.633)	0.107 (0.090, 0.137)	−0.511	<0.001

* OD_550_, optical density at 550 nm; data shown in median (interquartile range, IQR);

** R, resistance; I, intermediate; S, susceptible.

### Strong and weak biofilms provided similar levels of enhancement in antibiotic resistance

**Figure 4:**
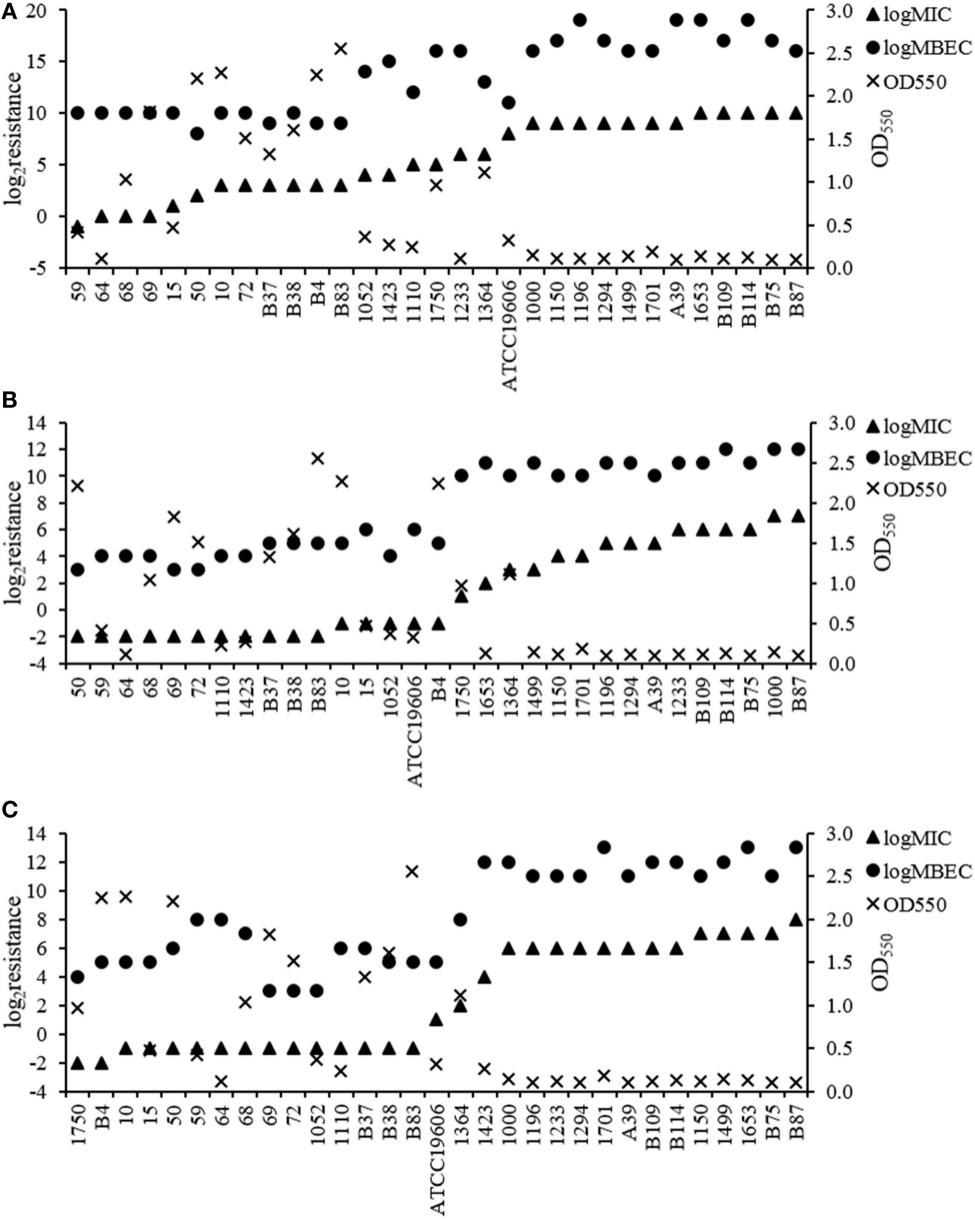
**Resistance in *A. baumannii* to cefotaxime, imipenem, and ciprofloxacin during planktonic and biofilm growth. (A)** Cefotaxime. **(B)** Imipenem. **(C)** Ciprofloxacin. For these three antibiotics, biofilm-specific resistance (MBEC) was consistently higher in isolates with higher antibiotic resistance of planktonic bacteria (MIC). The enhancement in resistance after biofilm formation occurred independent of biofilm quantity.

To assess whether biofilm-specific resistance is dependent on the quantity of biofilm biomass produced and/or on antibiotic resistance in planktonic mode, we determined the MBECs of cefotaxime, imipenem and ciprofloxacin for the sampled 31 *A. baumannii* isolates. The MICs of cefotaxime for these isolates ranged from 0.5 to 1024 μg/mL (Figure 4). Additionally, the MBECs of cefotaxime for these isolates were as high as 256–524,288 μg/mL, which was 8–2048-fold higher than their respective MIC values. For imipenem, the MIC ranged from 0.25 to 128 μg/mL and MBECs ranged from 8 to 4096 μg/mL, 32-512-fold higher than their respective MIC values. For ciprofloxacin the MICs ranged from 0.25 to 256 μg/mL and MBECs ranged from 8 to 8192 μg/mL, 16-512-fold higher than their respective MIC values.

We first analyzed the correlation between MIC values of the three antibiotics and the biofilm-forming capacities of the 31 strain and found negative correlation between the two qualities (*r*_s_ = −0.713 − −0.789, *P* < 0.001), further indicating the inverse relationship between biofilm formation and resistance to even one antibiotic. Subsequently, by assessing the relationship between MIC and MBEC values, we detected a positive correlation between antibiotic resistance (MIC values) and biofilm-specific resistance (MBEC values) for the three antibiotics (*r*_s_ = 0.844 − 0.921, *P* < 0.001). As depicted in Figure 4, there was a similar level of enhancement in resistance after biofilm formation among the majority of the isolates tested. Spearman's rank correlation analysis indicated that for each of the three antibiotics, this enhancement in resistance occurred independent of the level of biomass produced (*r*_s_ = −0.263 − 0.295, *P* > 0.05).

## Discussion

Antibiotic resistance in *A. baumannii* is an issue of increasing concern. In this study, the prevalence of MDR and XDR isolates was ~72.4%. These isolates also accounted for the main epidemic clusters detected. Non-MDR population did not form into clusters containing more than two isolates (Figure S1). The rate of carbapenems resistance in *A. baumannii* is also increasing during the past two decades in China. In the period of 1993–2003, the reported rate was 5%, while by 2004, the rate increased dramatically to 20–50% (Wang et al., 2007), and in this study, we found the carbapenems resistance rate was up to 60%. Furthermore, the prevalence of biofilm-forming strains among the tested *A. baumannii* isolates was greater than 91%. These data emphasized the importance of further research to develop treatments against *A. baumannii* infections.

Here, we analyzed the antibiotic resistance and biofilm forming potential of *A. baumannii* strains, and came to several findings. First, the population that exhibited more robust biofilm formation likely contained larger proportion of non-MDR isolates. Second, the MDR and XDR isolates, though belonging to different PFGE clusters, tended to form weaker biofilms than non-MDR strains. Third, we noticed special cases from PFGE analysis. For example, the only two non-MDR isolates in cluster9 were among the four strongest biofilm formers in this cluster and the one non-MDR isolate in cluster34 was also the strongest biofilm former in this cluster. Together, these results indicated that for *A. baumannii*, there was a statistically negative correlation between antibiotic resistance and biofilm forming capacity.

Rodriguez-Bano et al. previously reported that for *A. baumannii*, biofilm-forming isolates were less frequently resistant to imipenem and ciprofloxacin, indicating that these strains are not as dependent on antimicrobial resistance as non-biofilm-forming strains for survival (Rodríguez-Baño et al., 2008) which is consistent with our result. Moreover, exposure to sub-MIC levels of certain antibiotics promotes biofilm formation of *Staphylococcus aureus*, indicating that biofilms tend to be stronger when resistance is challenged (O'Neill et al., 2008; Kaplan, 2011). In this study, biofilm-forming capacity was measured in the absence of antibiotic-mediated stress. Therefore, our findings indicate that in *A. baumannii*, more susceptible isolates inherently tended to produce stronger biofilms. Together, we think biofilm acts as a mechanism for bacteria to get a better survival, especially in cases of when resistance level is not high enough. While the mechanisms that govern this process are not clear yet, expression of the β-lactamase gene *bla*_TEM−1_ is known to inhibit biofilm formation of *P. aeruginosa* by perturbing cell adhesion, thereby establishing a genetic link between biofilm production and antimicrobial resistance (Gallant et al., 2005). The presence of plasmids was also known to be associated with both antibiotic resistance and biofilm formation. They could enhance the ability of transferring resistance markers by transformation or conjugation (Sherley et al., 2004). Meanwhile, genes encode the protein of flagella and fimbriae are also located in plasmids. These two structures could facilitate biofilm formation (Karch et al., 1987). In this study, we found isolates with higher level of resistance always harbored more plasmids. But no obvious difference in biofilm formation was observed among strains with different plasmid profiles. Further analyses including detailed plasmid map are needed to figure out the influence of plasmid on the relationship between these two capacities. Explorations of beta-lactamase activity in different conditions and find genetic links between biofilm and antibiotic resistance other than *bla*_TEM−1_ are required to fully elucidate the mechanisms involved in these processes.

The analyses of the MICs and MBECs were also consistent with previous reports showing that biofilm population exhibit enhanced antimicrobial resistance compared to planktonic populations (Cernohorská and Votava, 2004). Specifically, our results demonstrated that most of the *A. baumannii* isolates (30/31) within biofilms exhibited 64–2048-fold greater resistance to cefotaxime than those under planktonic growth conditions. For imipenem and ciprofloxacin, the resistance could increase 32–512-fold and 16–512-fold, respectively. More importantly, we found that the increase in resistance of the three antibiotics occurred independent of the quantity of biofilm biomass produced. Previous studies demonstrated that mutations within the *ndvB* gene of *P. aeruginosa*, which encodes a glucosyltransferase, resulted in increased sensitivity of *P. aeruginosa* biofilms to several antibiotics without affecting the biofilm-forming capacity of the organism (Mah et al., 2003; Zhang et al., 2013). Spoering and Lewis (2001) suggested that more persisters led to greater resistance in *P. aeruginosa* biofilms. These findings genetically indicate that biofilm-specific resistance could be regulated independent of biofilm quantity, which is consistent with our results. In fact, the positive correlation detected between the MIC and MBEC values suggest that biofilm-specific resistance is primarily dependent on the level of antibiotic resistance of the organism and that the enhancement may be mainly the consequence of persisters and/or genetic factors described above. This is worth-noticing because for MDR/XDR isolates, even though their biofilms are always quite weak, they could still get a huge enhancement in resistance after biofilm formation.

In conclusion, although there are individual differences among the isolates, the results in this study indicate the existence of correlation between antibiotic resistance, biofilm formation, and biofilm-specific resistance statistically in *A. baumannii*. The findings raise questions regarding the mechanisms through which bacteria maintain a balance between biofilm formation capacity and antibiotic resistance, as well as how resistant strains achieve high levels of biofilm-specific resistance despite producing weak biofilms. Deeper explorations of plasmid maps and genetic regulation, such as identification of genes involved in biofilm-specific resistance and persisters, would improve our understanding of these processes. Clarifying these mechanisms could provide novel insights that would facilitate the development of therapeutics and prevention against *A. baumannii* biofilm-related infections.

## Author contributions

LQ carried out the biofilm formation and resistance studies, participated in the identification of the isolates and drafted the manuscript. HL participated in the data analysis and helped drafting the manuscript. CZ and BL participated in the identification and resistance tests of the isolates. JL, LW, and XD carried out the identification of the isolates. XL participated in its design and coordination. SQ and HS conceived of the study, and participated in its design and coordination and helped to draft the manuscript. All authors read and approved the final manuscript.

## Funding

This work was supported by the Mega-projects of Science and Technology Research (no. 2013ZX10004607), the National Nature Science Foundation of China (nos. 81373053 and 81371854), and the Beijing Science and Technology Nova program (no. xx2013061).

### Conflict of interest statement

The authors declare that the research was conducted in the absence of any commercial or financial relationships that could be construed as a potential conflict of interest.
