# Supplementary figures and images for: Relationship between Antibiotic Resistance, Biofilm Formation, and Biofilm-Specific Resistance in Acinetobacter baumannii
Source: Front Microbiol. 2016 Apr 12;7:483. doi: 10.3389/fmicb.2016.00483 (PMC4828443; doi:10.3389/fmicb.2016.00483)

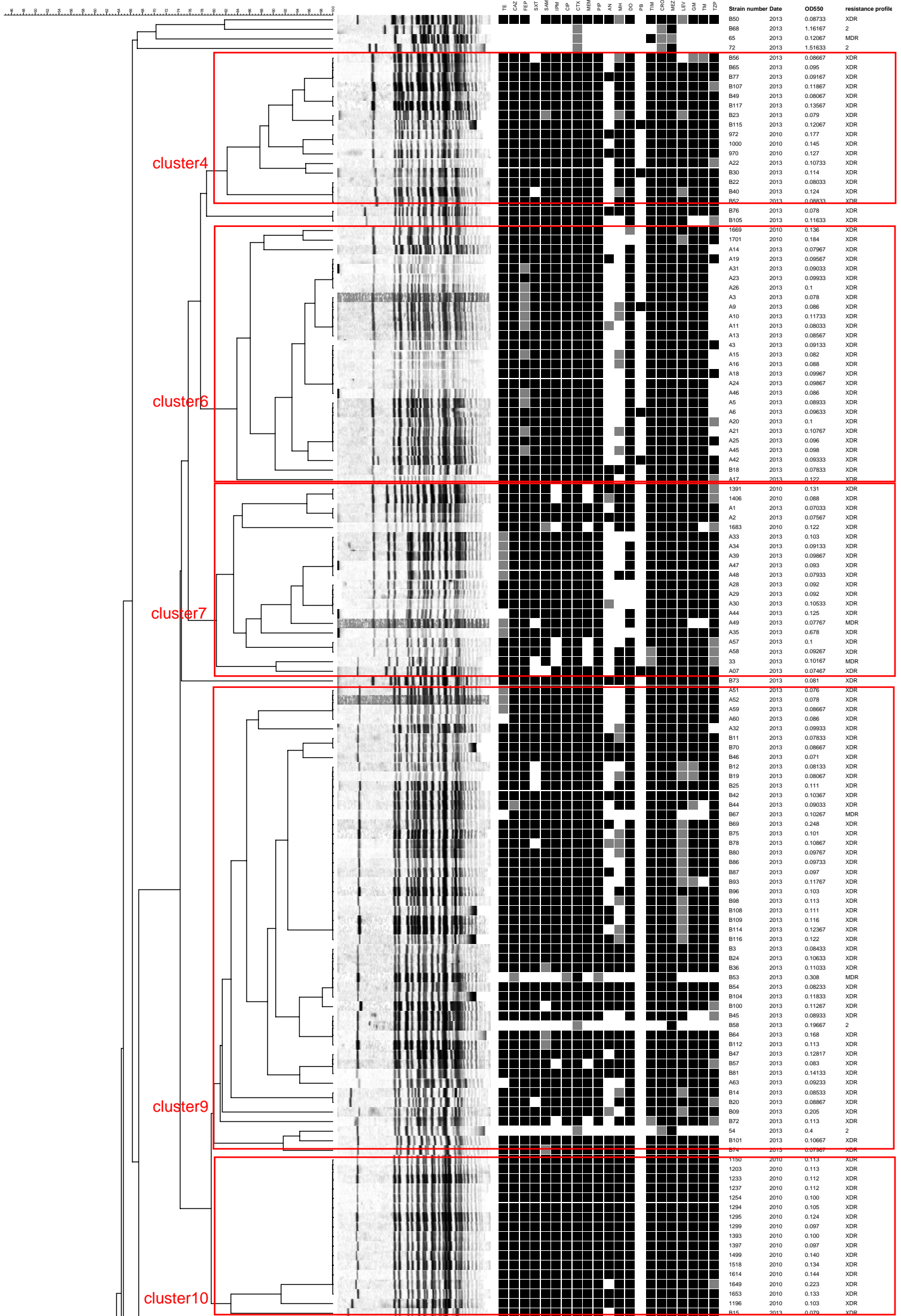

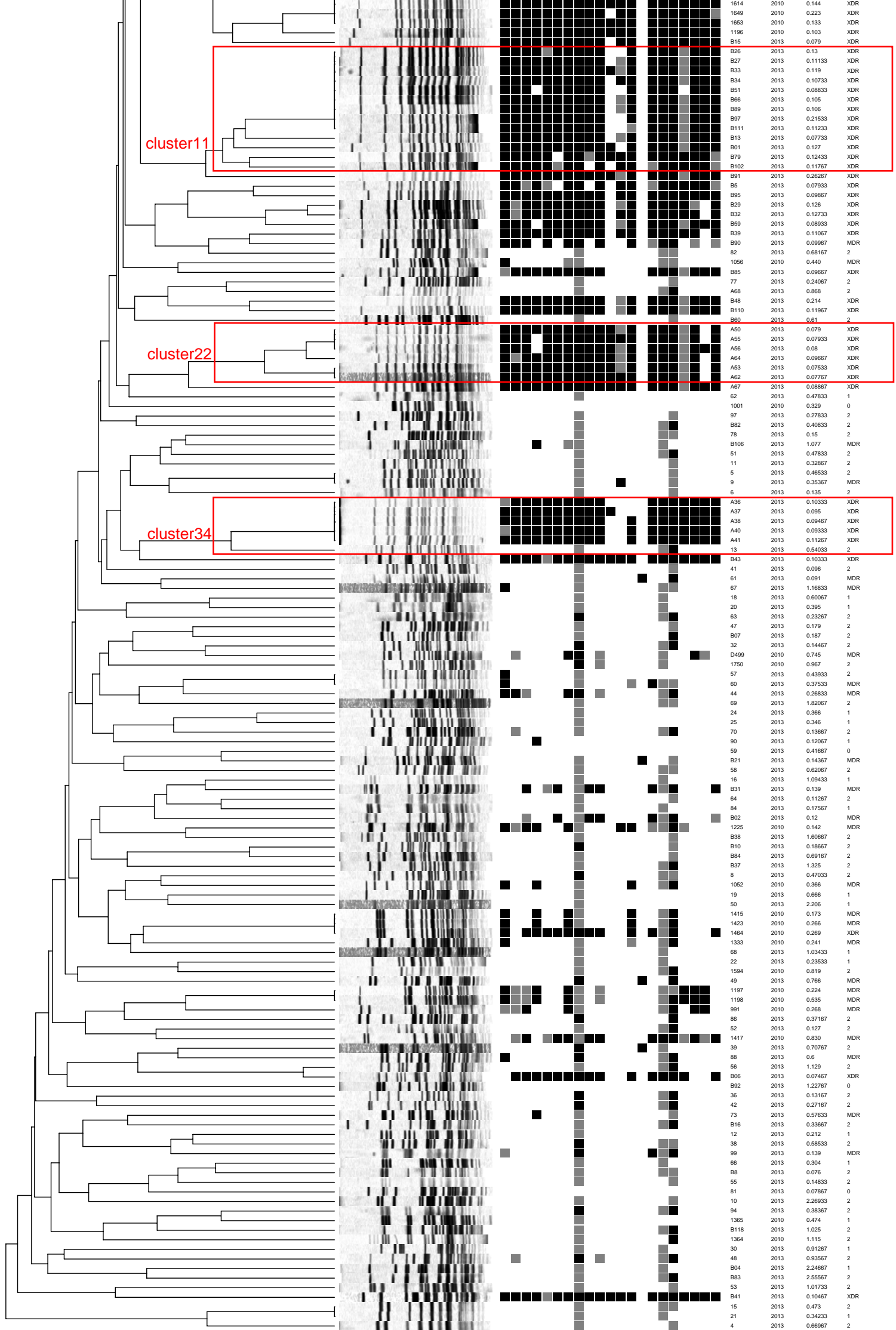

Supplement: Supplementary file 1 [file Image1.PDF]

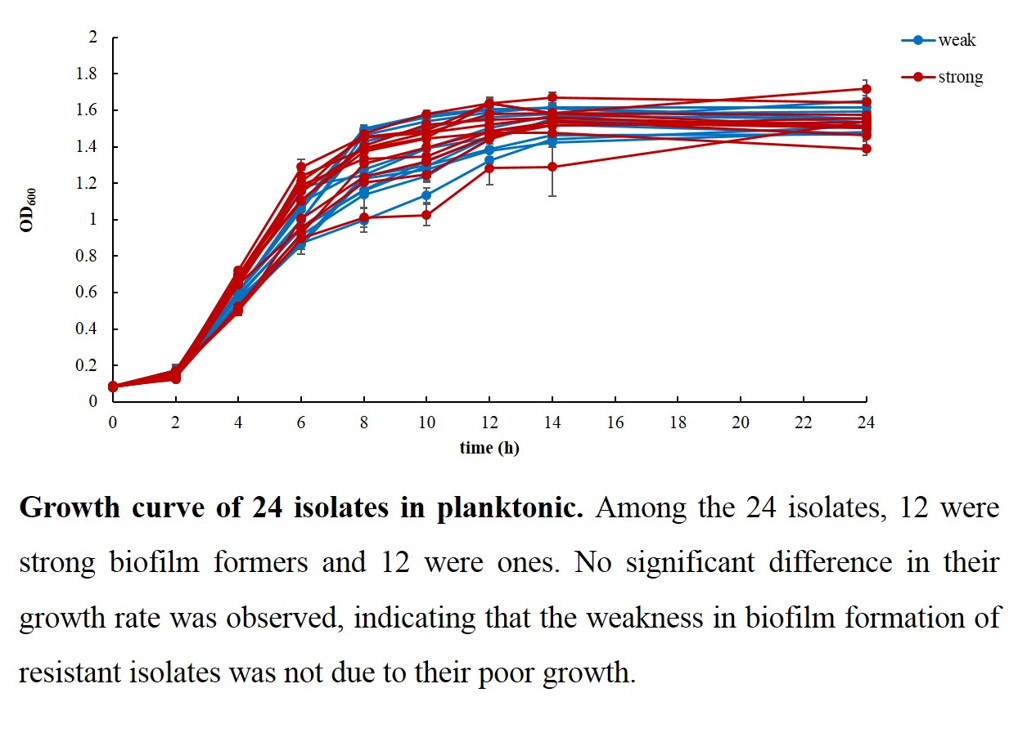

Supplement: Supplementary file 3 [file Image3.TIF]
