# Supplementary material for: Relationship between Antibiotic Resistance, Biofilm Formation, and Biofilm-Specific Resistance in Acinetobacter baumannii
Source: Front Microbiol. 2016 Apr 12;7:483. doi: 10.3389/fmicb.2016.00483 (PMC4828443; doi:10.3389/fmicb.2016.00483)
Supplement: Supplementary file 2 [file Image2.PDF]

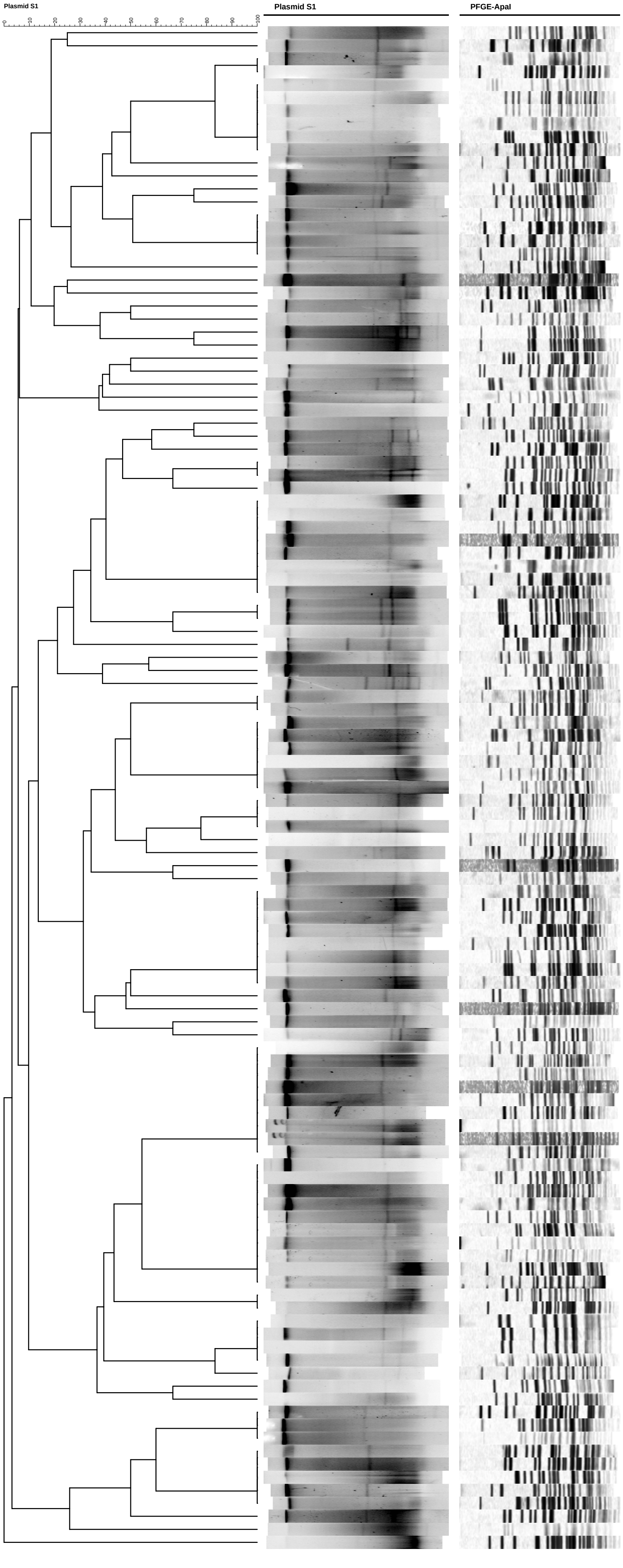

Strain number	resistance profile	n=	OD value
41	2	n=1	0.096
1225	MDR	n=1	0.142
94	2	n=1	0.384
97	2	n=2	0.278, 0.408
16	1	n=1	1.094
57	2	n=1	0.439
60	MDR	n=2	0.268, 0.375
64	2	n=1	0.113
66	1	n=1	0.304
B73	XDR	n=1	0.081
B111	XDR	n=9	0.121 (0.112, 0.124)
B118	2	n=1	1.025
24	1	n=1	0.366
1391	XDR	n=5	0.100 (0.097, 0.130)
33	MDR	n=1	0.102
B41	XDR	n=1	0.105
B43	XDR	n=1	0.103
B46	XDR	n=8	0.104 (0.091, 0.110)
B59	XDR	n=1	0.089
68	1	n=1	1.034
B60	2	n=1	0.610
991	MDR	n=1	0.268
A57	XDR	n=2	0.100, 0.093
1197	MDR	n=1	0.224
1198	MDR	n=1	0.535
12	1	n=1	0.212
58	2	n=1	0.621
84	1	n=1	0.176
A55	XDR	n=4	0.075, 0.079, 0.079, 0.080
B16	2	n=1	0.337
B38	2	n=1	1.607
D499	MDR	n=1	0.745
49	MDR	n=1	0.766
5	2	n=1	0.465
90	1	n=1	0.121
9	MDR	n=1	0.354
10	2	n=1	2.269
B83	2	n=1	2.556
13	2	n=1	0.540
39	2	n=1	0.708
81	0	n=1	0.079
99	MDR	n=1	0.139
B04	1	n=1	2.247
1196	XDR	n=16	0.119 (0.110, 0.228)
1423	MDR	n=2	0.173, 0.266
1464	XDR	n=1	0.269
73	MDR	n=1	0.576
30	1	n=1	0.913
B06	XDR	n=1	0.075
1750	2	n=1	0.967
1417	MDR	n=1	0.830
B09	XDR	n=4	0.080, 0.107, 0.113, 0.205
B95	XDR	n=2	0.079, 0.099
20	1	n=1	0.395
53	2	n=1	1.017
59	0	n=1	0.417
B02	MDR	n=1	0.120
A64	XDR	n=3	0.078, 0.089, 0.097
B110	XDR	n=1	0.120
A07	XDR	n=1	0.075
B14	XDR	n=2	0.085, 0.089
B48	XDR	n=1	0.214
B15	XDR	n=1	0.079
B31	MDR	n=1	0.139
69	2	n=1	1.821
B22	XDR	n=22	0.109 (0.080, 0.126)
18	1	n=1	0.601
B90	MDR	n=1	0.100
56	2	n=1	1.129
65	MDR	n=1	0.121
B01	XDR	n=4	0.118, 0.124, 0.127, 0.263
1364	2	n=1	1.115
1365	1	n=1	0.474
B39	XDR	n=1	0.111
72	2	n=1	1.516
67	MDR	n=1	1.168
1594	2	n=1	0.819
1701	XDR	n=2	0.136, 0.184
36	2	n=1	0.132
B106	MDR	n=1	1.077
43	XDR	n=25	0.417 (0.179, 0.936)
50	1	n=1	2.206
54	2	n=1	0.400
B07	2	n=1	0.187
A35	XDR	n=10	0.099 (0.094, 0.103)
A49	MDR	n=1	0.078
B68	2	n=1	1.162
6	2	n=1	0.135
8	2	n=1	0.470
51	2	n=1	0.478
62	1	n=1	0.478
1052	MDR	n=1	0.366
1056	MDR	n=1	0.440
A36	XDR	n=5	0.093, 0.095, 0.095, 0.103, 0.123
A68	2	n=1	0.868
B21	MDR	n=1	0.144
B85	XDR	n=1	0.097
11	2	n=1	0.329
19	1	n=1	0.666
4	2	n=1	0.670
15	2	n=1	0.473
21	1	n=1	0.342
61	MDR	n=1	0.091
B13	XDR	n=1	0.077
22	1	n=1	0.235
25	1	n=1	0.346
86	2	n=1	0.372
B67	MDR	n=2	0.102, 0.308
B19	XDR	n=31	0.111 (0.087, 0.129)
38	2	n=1	0.585
47	2	n=1	0.179
1001	0	n=1	0.329
B37	2	n=1	1.325
B50	XDR	n=1	0.087
88	MDR	n=1	0.600
55	2	n=1	0.148
1333	MDR	n=1	0.241
